# Assessment of Psychological Comorbidities Among Noncommunicable Disease Patients in Rural and Urban Areas of a District in Northern India: A Community-Based Cross-Sectional Study

**DOI:** 10.7759/cureus.95779

**Published:** 2025-10-30

**Authors:** Adarsh Maurya, Akansha Bansal, Urvashi Mavi, Pankaj K Jain, Dhiraj K Srivastava, Arun K Mishra, Anshul Kumar, Sushil K Shukla

**Affiliations:** 1 Community Medicine, Uttar Pradesh University of Medical Sciences, Etawah, IND; 2 Preventive Medicine, Uttar Pradesh University of Medical Sciences, Etawah, IND; 3 Psychiatry, Uttar Pradesh University of Medical Sciences, Etawah, IND

**Keywords:** anxiety, depression, non-communicable disease, psychological comorbidities, rural, stress, urban

## Abstract

Introduction: Psychiatric comorbidities such as depression, anxiety, and stress are two to threefold higher among patients with noncommunicable diseases (NCDs). These comorbidities are common in individuals with chronic NCDs and are linked to increased mortality and poorer health outcomes. Psychiatric disorders in patients with physical ailments are more likely to be associated with poor lifestyle choices and noncompliance with therapy, greater treatment costs, poor quality of life, deteriorating physical health, absenteeism from work, more hospitalizations, poor self-care, and increased mortality.

Aim and objectives: This article aims to assess psychological comorbidities among patients with noncommunicable diseases in rural and urban areas of Etawah, a district in northern India.

Materials and methods: A community-based cross-sectional study was conducted in both rural and urban areas of Etawah between October 2022 and March 2024. The study included 600 adults aged 18 years and above, of either gender, with one or more noncommunicable diseases. Participants were recruited through a multistage random sampling process. The study subjects were assessed using Patient Health Questionnaires (PHQs) for psychological comorbidities. The university’s ethical committee granted approval before the start of the study. IBM SPSS Statistics for Windows, Version 24 (Released 2016; IBM Corp., Armonk, New York), was used for statistical analysis after the data from the selected study units were entered into the SPSS spreadsheet.

Results: Of the 600 study subjects, 306 had at least one psychological comorbidity, accounting for a total prevalence of 51.0%. The prevalence was higher in the urban areas (55.3%) compared with the rural areas (46.7%), with a statistically significant difference (p = 0.034). Stress was the most common psychological comorbidity (50.0%), while anxiety and depression were present in 7.2% and 8.5% of study subjects, respectively.

Conclusion: There was a much higher burden of psychological comorbidities, mainly stress, affecting more than half of the study subjects. In comparison, anxiety and depression were observed more frequently among patients with cardiovascular diseases (CVDs), chronic kidney diseases (CKDs), and cancers. The burden of psychological comorbidities was significantly higher in urban areas than in rural ones. Therefore, more research is required to evaluate the factors that predict psychological comorbidities among NCD patients in rural and urban areas to alleviate the burden.

## Introduction

Noncommunicable diseases (NCDs) represent a significant and multifaceted category of health conditions that have garnered global attention due to their increasing prevalence, impact on well-being, and socioeconomic implications [1-3]. According to the World Health Organization (WHO), NCDs are conditions that are not caused by infectious agents and cannot be transmitted directly from person to person. The WHO (2023) estimates that NCDs result in the deaths of 41 million people annually and are responsible for 71% of all deaths globally. More than three-fourths of all NCD deaths (31.4 million, or 86%) occur in low- and middle-income countries, where NCDs disproportionately afflict people [1]. Nearly 5.8 million Indians (WHO report, 2015) lose their lives to NCDs such as cancer, diabetes, heart and lung illnesses, and stroke each year; that is, one in four Indians are at risk of dying from an NCD before turning 70 [4].

With the rise in NCDs, an increase in psychological morbidities has also been observed. Psychological morbidities, also known as mental health morbidities, refer to various mental and emotional disorders that affect an individual’s psychological well-being and overall functioning. Psychiatric comorbidities such as depression, anxiety, and stress are two- to threefold higher among NCD patients. A survey conducted in 60 countries by the WHO discovered that comorbid depression was present in 9.3% to 23.0% of patients with chronic NCDs. Collectively, psychological comorbidities are responsible for 72% of all NCD deaths [5].

In a study conducted in a South Indian city, somatization (35.1%), anxiety (19.1%), and depression (20.1%) were the psychological disorders identified [6]. About 35.5% of participants in another study conducted in a rural area of northern India suffered from depression, while 29% had anxiety disorders [7]. These findings indicate notable rural-urban disparities. These disorders can lead to significant distress, impair daily life, and may require treatment and support for effective management [8]. Psychological comorbidities can significantly diminish the quality of life for people with NCDs. Mental health challenges can affect adherence to treatment plans, exacerbate symptoms, and hinder overall well-being [6]. When combined with limited resources, the substantial burden of depression, anxiety, and stress that coexist with chronic physical diseases represents a serious public health concern for low- and middle-income nations [1].

Studying psychological comorbidities in NCDs is therefore crucial due to their significant impact on overall health, well-being, and healthcare outcomes. To date, no study has been conducted among both rural and urban populations of the same region. This study aims to assess the psychological comorbidities among patients with NCDs in rural and urban areas of the Etawah district. The primary objective of the study is to estimate the prevalence of psychological comorbidities among patients with NCDs, while the secondary objectives are to assess their severity and explore rural-urban disparities.

## Materials and methods

An urban- and rural-community-based cross-sectional survey was conducted in the district of Etawah between October 2022 and March 2024. The study subjects were NCD patients aged 30 years or older, of either gender, with one or more noncommunicable diseases such as diabetes mellitus, hypertension, cardiovascular diseases (e.g., CAD, CHF, MI), chronic pulmonary diseases (e.g., COPD, asthma), chronic kidney disease (CKD), or cancer (oral, breast, or cervical) for at least six months from the time of diagnosis, as confirmed by a registered medical practitioner (RMP). Participants who did not provide consent, had a known psychiatric disorder other than depression, anxiety, or stress, or were bedridden (unable to respond) were excluded.

To calculate the sample size, the following formula was used:

$n= (Z₁₋&alpha;̸̸ ₂)^{2}{P₁ (1- P₁) + P₂ (1- P₂)}/d^{2}$

where n is the sample size; Z₁₋α/₂ is the standard normal deviate (two-tailed), taken as 1.96 at α = 0.05 and a 95% confidence level. Taking the prevalence of psychological morbidities in individuals aged 30 years or older in rural (P₁) and urban (P₂) areas as 0.29 [7] and 0.19 [6], respectively, d was the margin of error on either side of the proportion, taken as 0.07. Applying the formula yielded n = 281; hence, the rounded sample size was 300 for each group. The final sample size was 600, comprising 300 participants each from the rural and urban areas of the Etawah district.

The target population was selected using a multistage random sampling technique from wards and blocks in the Etawah district, Uttar Pradesh. In the first stage, four blocks and eight wards were selected randomly. In the second stage, two villages were chosen using simple random selection from the list of all villages in each selected block to maintain equal representation (a total of eight villages were selected, two from each block), and one colony was chosen from each selected ward (a total of eight colonies). Standing in front of a landmark (temple, school, etc.), the surveyor turned to the left, and the first house in that direction was taken as the starting point of the survey. Data were collected from study subjects in each selected village and colony through systematic random sampling by house-to-house visits, continuing until the required sample size was achieved. In each household, the study subject was selected per the inclusion criteria. If any family had more than one eligible participant, one was selected randomly by the lottery method at the time of visit. If the required number of study subjects could not be obtained from a selected village or colony, selection continued in an adjacent village or colony until the target number was reached. If no eligible subject was available in a selected house or consent was not given, the next household was approached. Study subjects were informed in detail about the purpose of the project, and formal written consent was obtained. Information regarding sociodemographic profile, noncommunicable disease profile, and psychological comorbidities (using Patient Health Questionnaires) was collected as described in the study tool.

Study tool

A standardized, pretested, and predesigned questionnaire was used for interviews. It comprised three domains: sociodemographic profile, clinical profile, and Patient Health Questionnaires (PHQ-15 for stress, GAD-7 for anxiety, and PHQ-9 for depression) [9,10]. For each study participant, a separate questionnaire was completed. A pretest (pilot study) was conducted in the field practice areas of the Department of Community Medicine, Uttar Pradesh University of Medical Sciences (UPUMS), Saifai, namely Ujahyani and Bhaguiya. Ten percent of the study participants were selected for the pilot study. The questionnaires were reviewed for completeness, and modifications were made to ensure clarity in the local language (Hindi). For validity, the tool was evaluated through expert feedback and pretesting. Reliability was assessed by translating the questionnaire into local dialects, and Cronbach’s alpha was found to be greater than 0.7, indicating acceptable internal consistency.

Ethical considerations

The university’s ethics committee granted approval before the start of the study (Ref. No. 505/UPUMS/DSW/Ethical/2022-23, dated December 22, 2022; ethical clearance number 56/2022-23). Before conducting the survey, written informed consent was obtained from each participant after an explanation of the study’s purpose, nature, and procedure in their native language. Participants were assured that confidentiality, privacy, and anonymity would be strictly maintained at all levels. Withdrawal from the study was permitted at any stage without conditions. Prior training was obtained from experts in the Department of Psychiatry, UPUMS, for screening and diagnosis of different psychological comorbidities to maintain quality control. Patients screened with psychological comorbidities were counseled and advised to visit the Department of Psychiatry, UPUMS, Saifai.

Data entry and statistical analysis

For identification, each family was assigned a unique code noted in the interview forms. The supervisor and co-supervisor cross-checked the collected data for completeness and accuracy. IBM SPSS Statistics for Windows, version 24 (Released 2016; IBM Corp., Armonk, New York), was used for statistical analysis after the data gathered from the selected study units were entered into the SPSS spreadsheet. The findings were presented in tables and graphs with percentages, interquartile ranges, means, medians, standard deviations, and proportions using appropriate statistical tests such as Fisher’s exact test and Pearson’s chi-square test. A 95% confidence interval and a p-value less than 0.05 were considered statistically significant.

## Results

The study comprised 600 individuals (Table 1) with a mean age of 55.52 (±12.17) years, predominantly from the 40-49 and 60-69 age groups (157 participants, 26.2% each). In rural areas, the 60-69 age group was most prevalent (81, 27.0%), while in urban areas, the 40-49 age group predominated (88, 29.3%). Gender distribution was nearly equal, with 315 females (52.3%), consistent across rural (158, 52.7%) and urban (157, 52.3%) settings. Religious affiliation was predominantly Hindu (569, 94.8%), with rural (289, 96.3%) and urban (280, 93.3%) areas showing similar patterns. Caste distribution revealed that 296 (49.4%) participants belonged to other backward classes (OBCs), with rural (140, 46.7%) and urban (156, 52.0%) populations showing comparable proportions. Statistically significant variations were noted in marital status (p = 0.002), occupation (p = 0.045), education (p < 0.001), family type (p = 0.004), and socioeconomic class (p = 0.005) between rural and urban populations. Most participants were married (531, 88.5%) and belonged to joint families (264, 60.7%). Educationally, 183 (30.5%) were illiterate, with urban areas (106, 35.3%) showing higher illiteracy rates than rural areas (77, 25.3%). Occupationally, 288 (48.0%) were homemakers, with rural (141, 47.0%) and urban (147, 49.0%) areas displaying similar distributions. Socioeconomically, 586 (97.7%) were classified as lower class, with rural (299, 99.7%) and urban (287, 95.7%) areas differing slightly.

**Table 1:** Sociodemographic characteristics of study subjects in rural and urban areas of Etawah (N = 600) ^1^Others include Sikh, Jain, and Christian. ^2^ Others include widow/widower, divorced, separated. ^$^There were no research participants in the upper class or upper middle class according to the revised BG Prasad classification (as per AICPI January 2024), so these two categories are not mentioned in socioeconomic class in this table.

Variables	Total (N=600)	Rural (N=300)	Urban (N=300)
	n (%)	n (%)	n (%)
Age groups (in years) (Mean ± SD: 55.52 ± 12.177)			
30-39	52(8.6)	22(7.3)	30(10.0)
40-49	157(26.2)	69(23.0)	88(29.3)
50-59	135(22.5)	74(24.7)	61(20.3)
60-69	157(26.2)	81(27.0)	76(25.3)
≥70	99(16.5)	54(18.0)	45(15.0)
Gender			
Male	285(47.5)	142(47.3)	143(47.7)
Female	315(52.5)	158(52.7)	157(52.3)
Religion			
Hindu	569(94.8)	289(96.3)	280(93.3)
Muslim	30(5.0)	11(3.7)	19(6.3)
Others^1^	1(0.2)	-	1(0.3)
Caste			
General	167(27.8)	92(30.7)	75(25.0)
OBC (other backward class)	296(49.4)	140(46.7)	156(52.0)
SC/ST (schedule caste/ schedule tribe)	137(22.8)	68(22.7)	69(23.0)
Marital status			
Unmarried	19(3.2)	8(2.7)	11(3.7)
Married	531(88.5)	276(92.0)	255(85.0)
Others^2 ^	50(8.3)	16(5.3)	34(11.3)
Type of family			
Single-member family	5(0.8)	-	5(1.7)
Nuclear	231(38.5)	103(34.3)	128(42.7)
Joint	364(60.7)	197(65.7)	167(55.7)
Educational status			
Illiterate	183(30.5)	77(25.7)	106(35.3)
Primary school	143(23.8)	68(22.7)	75(25.0)
Middle school	76(12.7)	43(14.3)	33(11.0)
Highschool	86(14.3)	56(18.7)	30(10.0)
Intermediate	34(5.7)	24(8.0)	10(3.3)
Graduate	51(8.5)	27(9.0)	24(8.0)
Postgraduate	27(4.5)	5(1.7)	22(7.3)
Occupation			
Unemployed	68(11.3)	43(14.3)	25(8.3)
Government employee	34(5.7)	11(3.7)	23(7.7)
Non-government employee	59(9.8)	25(8.3)	34(11.3)
Self-employed	130(21.7)	70(23.3)	60(20.0)
Homemaker	288(48.0)	141(47.0)	147(49.0)
Retired/pensioners	21(3.5)	10(3.3)	11(3.7)
Socioeconomic class of family (Modified B J Prasad Classification)^$^			
Lower middle class	1(0.2)	-	1(0.3)
Upper lower class	13(2.2)	1(0.3)	12(4.0)
Lower class	586(97.7)	299(99.7)	287(95.7)
Monthly expenditure on treatment (INR) (median (IQR)) (600 (200, 2000))			
Nil	89(14.8)	43(14.3)	46(15.3)
0-5,000	464(77.3)	235(78.3)	229(76.3)
5,001-10,000	29(4.8)	10(3.3)	19(6.3)
>10,000	18(3.0)	12(4.0)	6(2.0)
Catastrophic health expenditure			
Yes	81(13.8)	46(15.3)	35(11.3)
No	519(86.5)	254(84.7)	265(88.7)

The study assessed participants’ psychological comorbidities among six major NCDs (Table 2). Hypertension was the most prevalent (343, 57.2%), followed by diabetes (228, 38.0%), chronic pulmonary diseases (124, 20.0%), and cardiovascular diseases (69, 11.5%). Rural and urban areas exhibited similar disease patterns. Chronic kidney disease and cancer were less common, affecting 15 (2.5%) and 9 (1.5%) participants, respectively. Most participants (432, 72.0%) had only one NCD, with rural (207, 69.0%) and urban (225, 75.0%) areas showing similar patterns. Only 20 (3.3%) participants had more than two NCDs. The majority (259, 43.2%) had been ill for one to five years, with rural (141, 47.0%) and urban (118, 39.3%) areas differing slightly. Asymptomatic individuals comprised 328 (54.7%) of the study population.

**Table 2:** Clinical profile of study subjects in rural and urban areas of Etawah (N = 600) ^$^Multiple choices. Hence, the total will be more than 600.

Variables	Category	Total (N = 600), n (%)	Rural (N = 300), n (%)	Urban (N = 300), n (%)
Noncommunicable diseases^$^				
Diabetes mellitus	Yes	228(38.0)	122(40.7)	106(35.3)
	No	372(62.0)	178(59.3)	194(64.7)
Hypertension	Yes	343(57.2)	175(58.3)	168(56.0)
	No	257(42.8)	125(41.7)	132(44.0)
Cardiovascular diseases (other than hypertension)	Yes	69(11.5)	35(11.7)	34(11.3)
	No	531(88.5)	265(88.3)	266(88.7)
Chronic pulmonary diseases	Yes	124(20.7)	55(18.3)	69(23.0)
	No	476(79.3)	245(81.7)	231(77.0)
Chronic kidney diseases	Yes	15(2.5)	9(3.0)	6(2.0)
	No	585(97.5)	291(97.0)	294(98.0)
Cancer	Yes	9(1.5)	6(2.0)	3(1.0)
	No	591(98.5)	294(98.0)	297(99.0)
Number of noncommunicable diseases				
One		432(72.0)	207(69.0)	225(75.0)
Two		148(24.7)	84(28.0)	64(21.3)
>Two		20(3.3)	9(3.0)	11(3.7)
Duration of illness				
<1 year		146(24.3)	76(25.3)	70(23.3)
1-5 years		259(43.2)	141(47.0)	118(39.3)
6-10 years		118(19.7)	48(16.0)	70(23.3)
>10 years		77(12.8)	35(11.7)	42(14.0)
Current status of the disease				
Asymptomatic		328(54.7)	155(51.7)	173(57.7)
Symptomatic		272(45.3)	145(48.3)	127(42.3)

Table 3 presents the prevalence of different psychological comorbidities in the study subjects. Stress was the most common psychological comorbidity (300, 50.0%), while anxiety and depression were present in 43 (7.2%) and 51 (8.5%) participants, respectively. Most study subjects had no psychological comorbidities in both rural and urban areas, while 239 (39.8%) had only one psychological comorbidity. A total of 46 (7.7%) had two comorbidities, and 21 (3.5%) had all three.

**Table 3:** Prevalence of different types of psychological comorbidities among study subjects (N = 600) ^1^PHQ-15 to assess stress. ^2^GAD-7 to assess anxiety. ^3^PHQ-9 to assess depression.

Variables	Category	Total (N = 600), n (%)
Types of psychological comorbidities		
Stress^1^	Yes	300 (50.0)
	No	300 (50.0)
Anxiety^2^	Yes	43 (7.2)
	No	557 (92.8)
Depression^3^	Yes	51 (8.5)
	No	549 (91.5)
Numbers of psychological comorbidities		
No psychological comorbidities		294 (49.0)
Only one		239 (39.8)
Two		46 (7.7)
Three		21 (3.5)

**Figure 1:**
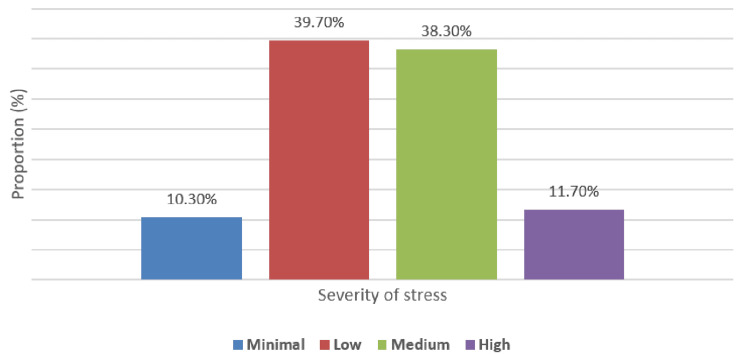
Distribution of severity of stress among study subjects (N = 300)

**Figure 2:**
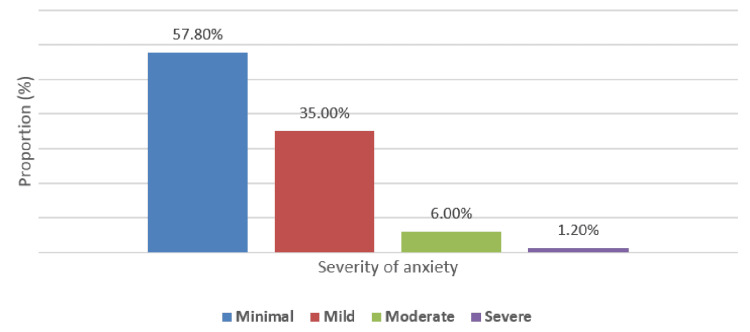
Distribution of severity of anxiety among study subjects (N = 43)

**Figure 3:**
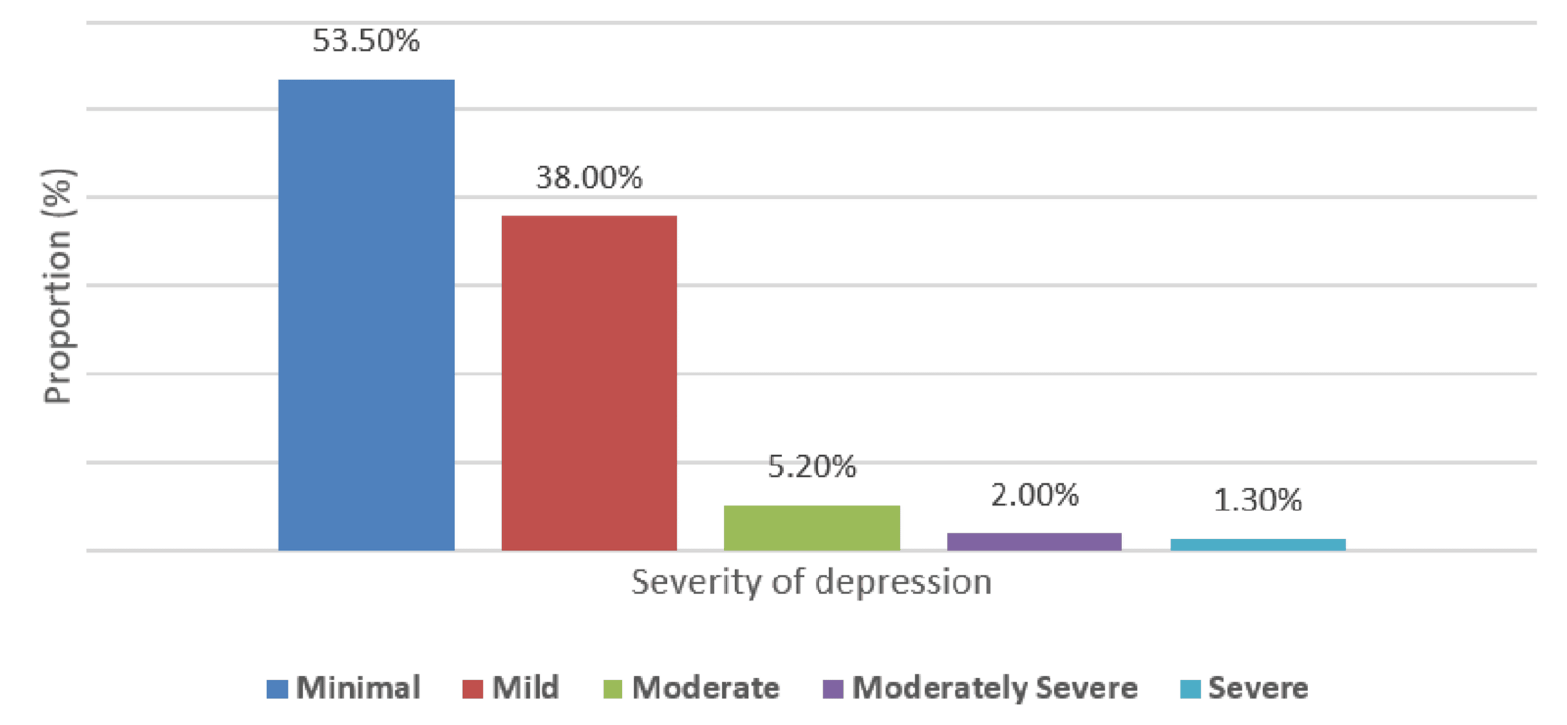
Distribution of severity of depression among study subjects (N = 51)

The severity of stress, anxiety, and depression is shown in Figures 1-3, respectively. Most participants had low (238, 39.7%) or moderate (230, 38.3%) stress severity; minimal and high stress were observed in 62 (10.3%) and 70 (11.7%), respectively. Most study subjects (347, 57.8%) had minimal anxiety, while only seven (1.2%) had severe anxiety. For depression, most participants (321, 53.5%) had minimal depression, and only eight (1.3%) had severe depression.

Table 4 presents the prevalence of overall psychological comorbidities among study participants from urban and rural regions. The prevalence was higher in urban areas (166, 55.3%) compared with rural areas (140, 46.7%), with a statistically significant difference (p = 0.034).

**Table 4:** Prevalence of psychological comorbidities among research participants in both urban and rural settings (N=300 each) ^1^PHQ (patient health questionnaires which included PHQ-15, GAD-7, and PHQ-9). Values are presented as (column percentage) (row percentage). ^#^Chi-square test. *Statistically significant association at 95% CI (p value < 0.05).

Psychological comorbidities ^1^	Rural (N = 300)	Urban (N = 300)	Chi-square value	Degree of freedom	p-value^ #^
	n (%)	n (%)			
Present	140 (46.7) (45.7)	166 (55.3) (54.3)	4.51	1	0.034*
Absent	160 (53.3) (54.4)	134 (44.7) (45.6)			

The frequency of various psychiatric comorbidities among participants in urban and rural settings is presented in Table 5. Stress was the most common psychological comorbidity among rural participants (138, 46.0%). In comparison, anxiety and depression were present in 19 (6.3%) and 25 (8.3%) participants, respectively. Stress was also the most common psychological comorbidity among urban participants (162, 54.0%), while depression and anxiety were present in 26 (8.7%) and 24 (8.0%) participants, respectively. Most participants with NCDs had no psychological comorbidities in both rural and urban areas, while 108 (36.0%) and 131 (43.7%) had only one psychological comorbidity in rural and urban areas, respectively. Twenty-four (8.0%) and 22 (7.3%) participants in urban and rural regions, respectively, had two comorbidities, while 11 (3.7%) and 10 (3.3%) participants in each region had all three. The distribution of these comorbidities did not differ significantly between rural and urban areas.

**Table 5:** Prevalence of different types of psychological comorbidities among study subjects in rural and urban areas (N = 300 each) ^1^PHQ-15 to assess stress. ^2^GAD-7 to evaluate anxiety. ^3^PHQ-9 to assess depression. ^#^Chi-square test. *Statistically significant association at 95% CI (p value < 0.05).

Variables	Category	Rural (N = 300), n (%)	Urban (N = 300), n (%)	Chi-square value	Degree of freedom	p-value^#^
Types of psychological comorbidities						
Stress^1^	Yes	138 (46.0)	162 (54.0)	3.841	1	0.050
	No	162 (54.0)	138 (46.0)			
Anxiety^2^	Yes	19 (6.3)	24 (8.0)	0.620	1	0.429
	No	281(93.7)	276 (92.0)			
Depression^3^	Yes	25 (8.3)	26 (8.7)	0.020	1	0.884
	No	275 (91.7)	274 (91.3)			
Number of psychological comorbidities						
No psychological comorbidities		160 (53.4)	134 (44.6)	3.217	3	0.200
Only one		108 (36.0)	131 (43.7)			
Two		22 (7.3)	24 (8.0)			
Three		10 (3.3)	11 (3.7)			

**Figure 4:**
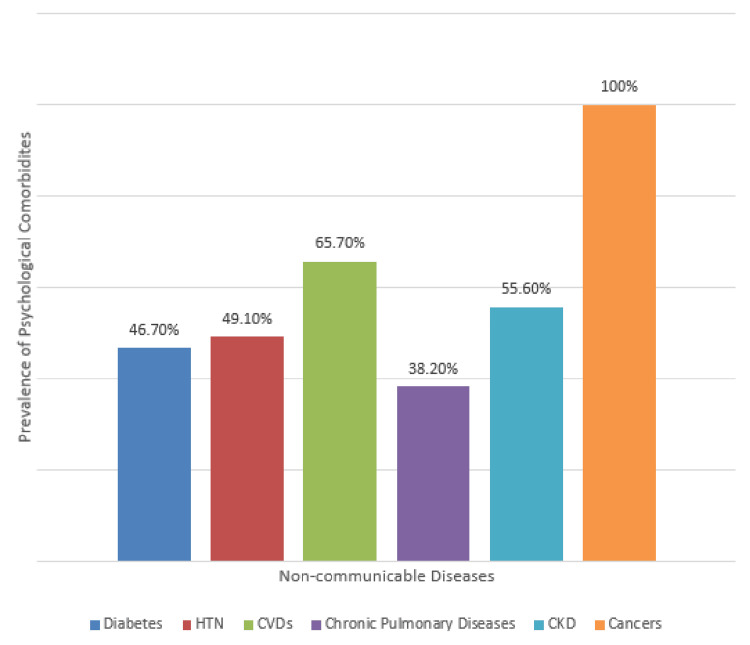
Prevalence of psychological comorbidities in study subjects having different noncommunicable diseases (N = 600) The figure provides a comparative picture of psychological comorbidities across all six noncommunicable diseases individually. For instance, among all diabetic study subjects who participated in the study, 46.7% had psychological comorbidities. All patients with cancer had some form of psychological comorbidity; however, this may not reflect the true picture because the total number of cancer patients in this study was very small. This was followed by patients with cardiovascular diseases, of whom 65.7% had psychological comorbidities. CVD: cardiovascular disease, CKD: chronic kidney disease, HTN: hypertension.

Figure 4 presents the distribution of psychological comorbidities among all study subjects with different NCDs. Psychological comorbidity was present in 106 (46.7%) participants with diabetes, 168 (49.1%) with hypertension, and 47 (38.2%) with chronic pulmonary diseases. Psychological comorbidity was present in more than half of participants with cardiovascular diseases (45, 65.7%) and chronic kidney disease (8, 55.6%). It was present in all participants with cancer (9, 100%).

## Discussion

According to the findings of this study, participants’ mean (±SD) age was 55.52 (±12.17) years, which was higher than that reported by Mehra et al. [7] and Kanwar et al. [11] in rural areas but similar to the findings of Kulkarni et al. [12] in an urban area. The majority of research participants were female (315, 52.3%), which was similar to Mehra et al. [7] but in contrast with Kulkarni et al. [12]. Similar to this study, Kulkarni et al. [12] and Mehra et al. [7] found that most participants (531, 88.5%) were married. In the present study, most participants were hypertensive, followed by diabetic, which contrasts with the findings of Sekhri et al. [13] in rural areas and Kulkarni et al. [12] in urban areas.

In this study, stress was the major psychological comorbidity (300, 50.0%) among the study subjects. In comparison, anxiety and depression were present in 43 (7.2%) and 51 (8.5%) participants, respectively, findings similar to those of Siddharthan et al. [14], Dare et al. [15], and Rajan et al. [16], where the prevalence of psychological comorbidities ranged from 3.9% to 44% in chronic NCDs. However, in contrast, Kanwar et al. [11] reported that 58.0% of participants suffered from mental comorbidity, with depression accounting for 41.9% of the total. In the present study, most participants had low to moderate stress severity, whereas Joseph et al. [17] found that most participants had moderate to severe psychological comorbidities. Similar to this study, stress was the major psychological comorbidity in urban areas, consistent with Kulkarni et al. [12], but in contrast to Mehra et al. [7], in which depression was predominant in rural areas. Although no statistically significant variation was observed in the distribution of these three comorbidities between rural and urban areas in this study, rural respondents had a higher prevalence of depression (57%) compared with urban respondents (31%), as reported by Thour et al. [18].

The strengths of this study include a representative sample of 600 participants. The analysis accounted for a structured survey design and included both rural and urban populations, providing insights into regional disparities. The use of validated tools, pilot testing, and intensive training minimized biases and errors, ensuring data quality. However, certain limitations exist. The equal representation of rural and urban areas may not reflect actual population proportions, potentially affecting generalizability. In addition, the relatively small number of participants with specific conditions such as cardiovascular diseases, chronic kidney disease, and cancer could lead to skewed outcomes.

## Conclusions

The study identified hypertension as the most prevalent NCD among participants, followed by diabetes, chronic pulmonary diseases, and cardiovascular diseases in the study area. There were no notable variations in the distribution of these diseases between rural and urban populations. Interestingly, there was a much higher burden of psychological comorbidities, mainly stress, affecting half of the study subjects. Anxiety and depression were higher only among patients with CVDs, CKD, and cancers in the present study. The burden of psychological comorbidities was significantly greater in urban regions than in rural ones.

Screening for psychological comorbidities should be promoted among all patients with NCDs visiting health facilities, as well as through community health workers, given the higher reported burden. After screening, confirmation by a psychiatrist should be followed by grievance redressal, support group formation, and appropriate management of both NCDs and psychological comorbidities. Further research should aim to identify the associated factors and predictors of psychological comorbidities in patients with NCDs to develop targeted interventions addressing these challenges.
